# Epigenetic Mechanisms Are Involved in Sex-Specific *Trans*-Generational Immune Priming in the Lepidopteran Model Host *Manduca sexta*

**DOI:** 10.3389/fphys.2019.00137

**Published:** 2019-03-04

**Authors:** Jasmin Gegner, Arne Baudach, Krishnendu Mukherjee, Rayko Halitschke, Heiko Vogel, Andreas Vilcinskas

**Affiliations:** ^1^Department of Bioresources, Fraunhofer Institute for Molecular Biology and Applied Ecology, Giessen, Germany; ^2^Institute for Insect Biotechnology, Faculty of Agricultural Sciences, Nutritional Sciences, and Environmental Management, Justus-Liebig University of Giessen, Giessen, Germany; ^3^Department of Entomology, Max Planck Institute for Chemical Ecology, Jena, Germany; ^4^Department of Molecular Ecology, Max Planck Institute for Chemical Ecology, Jena, Germany

**Keywords:** epigenetics, innate immunity, *trans*-generational immune priming, pathogens, *Manduca sexta*, *Serratia entomophila*

## Abstract

Parents invest in their offspring by transmitting acquired resistance against pathogens that only the parents have encountered, a phenomenon known as *trans*-generational immune priming (TGIP). Examples of TGIP are widespread in the animal kingdom. Female vertebrates achieve TGIP by passing antibodies to their offspring, but the mechanisms of sex-specific TGIP in invertebrates are unclear despite increasing evidence suggesting that both male-specific and female-specific TGIP occurs in insects. We used the tobacco hornworm (*Manduca sexta*) to investigate sex-specific TGIP in insects because it is a model host for the analysis of insect immunity and the complete genome sequence is available. We found that feeding larvae with non-pathogenic *Escherichia coli* or the entomopathogen *Serratia entomophila* triggered immune responses in the infected host associated with shifts in both DNA methylation and histone acetylation. Maternal TGIP was mediated by the translocation of bacterial structures from the gut lumen to the eggs, resulting in the microbe-specific transcriptional reprogramming of genes encoding immunity-related effector molecules and enzymes involved in the regulation of histone acetylation as well as DNA methylation in larvae of the F1 generation. The third-instar F1 larvae displayed sex-specific differences in the expression profiles of immunity-related genes and DNA methylation. We observed crosstalk between histone acetylation and DNA methylation, which mediated sex-specific immune responses in the F1 generation derived from parents exposed to a bacterial challenge. Multiple routes for TGIP seem to exist in *M. sexta* and – partially sex-specific – effects in the offspring depend on the microbial exposure history of their parents. Crucially, the entomopathogen *S. entomophila* appears to be capable of interfering with TGIP in the host.

## Introduction

Parents can invest in their offspring by preparing them to cope with pathogens or parasites that only the parents have encountered. The transfer of immunity from parents to offspring is known as *trans*-generational immune priming (TGIP) and has been reported in a wide range of animals, including arthropods (Little et al., 2003; Sadd et al., 2005; Dubuffet et al., 2015; Milutinović et al., 2016). The mechanisms underlying TGIP and the specificity of the resulting immune responses have been investigated in insects such as the bumblebee (*Bombus* spp.), the mealworm beetle (*Tenebrio molitor*), the red flour beetle (*Tribolium castaneum*), and lepidopterans such as the greater wax moth (*Galleria mellonella*) and the tobacco hornworm (*Manduca sexta*) (Dubuffet et al., 2015; Trauer-Kizilelma and Hilker, 2015b; Milutinović et al., 2016; Vilcinskas, 2016; Castro-Vargas et al., 2017; Rosengaus et al., 2017).

Mechanisms of parental investment in the form of TGIP differ between vertebrates and invertebrates and between sexes (Hasselquist and Nilsson, 2009; Roth et al., 2010; Herren et al., 2013; Eggert et al., 2014; Freitak et al., 2014; Knorr et al., 2015; Salmela et al., 2015), supporting Bateman’s principle that males gain fitness by increasing their mating success whereas females increase fitness through longevity because their reproductive effort is much higher (Rolff, 2002). The maternal transfer of immunity in vertebrates is realized by antibodies, which are provided by the mother during gestation and (in mammals) during lactation (Hasselquist and Nilsson, 2009). However, the mechanisms of maternal TGIP in insects were unclear until a recent report revealed that bacteria taken up with the diet can translocate from the larval gut to the hemocoel and are ultimately deposited in the developing eggs (Freitak et al., 2014), apparently by binding to egg-yolk proteins (Salmela et al., 2015). The *trans*-generational transmission of bacteria via yolk proteins has also been observed in *Drosophila melanogaster* (Herren et al., 2013). The transfer of bacteria or fragments thereof from mothers to eggs explains at least in part the specificity of maternal TGIP in *G. mellonella* and *T. castaneum* (Freitak et al., 2014;Knorr et al., 2015).

Paternal TGIP is also observed in insects, but the immunological protection is less specific than that conferred by maternal TGIP and the mechanism is unclear (Roth et al., 2010; Eggert et al., 2014). Current concepts in evolutionary biology postulate that environmental stimuli such as stress and pathogens can be translated into heritable phenotypic alterations by epigenetic mechanisms (Nestler, 2016). Therefore, epigenetic mechanisms may explain how fathers can also translate information about the pathogens they have encountered (environmental stimuli) into heritable adaptations of the offspring immune system (phenotypic alteration) without any genetic changes (Vilcinskas, 2016). There is also a large body of evidence indicating that pathogens influence epigenetic gene regulation in their insect hosts (Mukherjee et al., 2017; Vilcinskas, 2017).

We investigated the potential epigenetic basis of TGIP in insects using the tobacco hornworm *Manduca sexta* because male and female larvae are easy to distinguish morphologically, it is widely used as a lepidopteran model to study innate immunity (Jiang et al., 2010), and the complete genome sequence was published recently (Kanost et al., 2016). To mimic natural oral infections and to determine the pathogen-specificity of any TGIP we observed, we supplemented the larval diet with either non-pathogenic *Escherichia coli* or with the entomopathogen *Serratia entomophila*, both of which are known to translocate from the midgut into the hemocoel in *G. mellonella* (Freitak et al., 2014). We tracked bacteria added to the diet to determine whether they were transferred from the gut lumen to the eggs. We also monitored the infected larvae for evidence of an immune response in the host by looking for shifts in complex parameters, specifically developmental timing. We analyzed the expression profiles of selected immunity-related effector genes in F0 and F1 male and female larvae. To determine whether epigenetic mechanisms were suitable to analyze sex-specific TGIP effects we observed, we compared total DNA methylation and histone acetylation in the same cohorts. DNA methylation involves the addition of a methyl group to cytidine residues in the dinucleotide sequence CpG to form 5-methylcytidine, which retains the base-pairing capacity of the unmodified nucleotide but modifies its interaction with regulatory proteins (Vilcinskas, 2017) and seems to be associated with stably expressed genes, related to basic housekeeping in lepidopterans (Jones et al., 2018). *De novo* methylation is established by DNA methyltransferase 3 (DNMT3) and is maintained by the maintenance methyltransferase DNMT1. However, some insect taxa including the Lepidoptera have lost DNMT3 (Bewick et al., 2017). We therefore focused our analysis on DNMT1 and 2 and the methyl-CpG-binding domain protein (MBD). Similarly, the core histone proteins that combine with DNA to form chromatin can be modified to control the density of packing, with the removal of acetyl groups by histone deacetylases (HDACs) causing transcriptional repression due to the tighter packing and lack of access to the DNA and the addition of acetyl groups by histone acetyltransferases (HATs) having the opposite effect (Marks et al., 2003). Accordingly, we also looked at the relationship between histone modification and the expression of HATs and HDACs in both generations.

## Materials and Methods

### Insect Rearing and Diets

**FIGURE 1:**
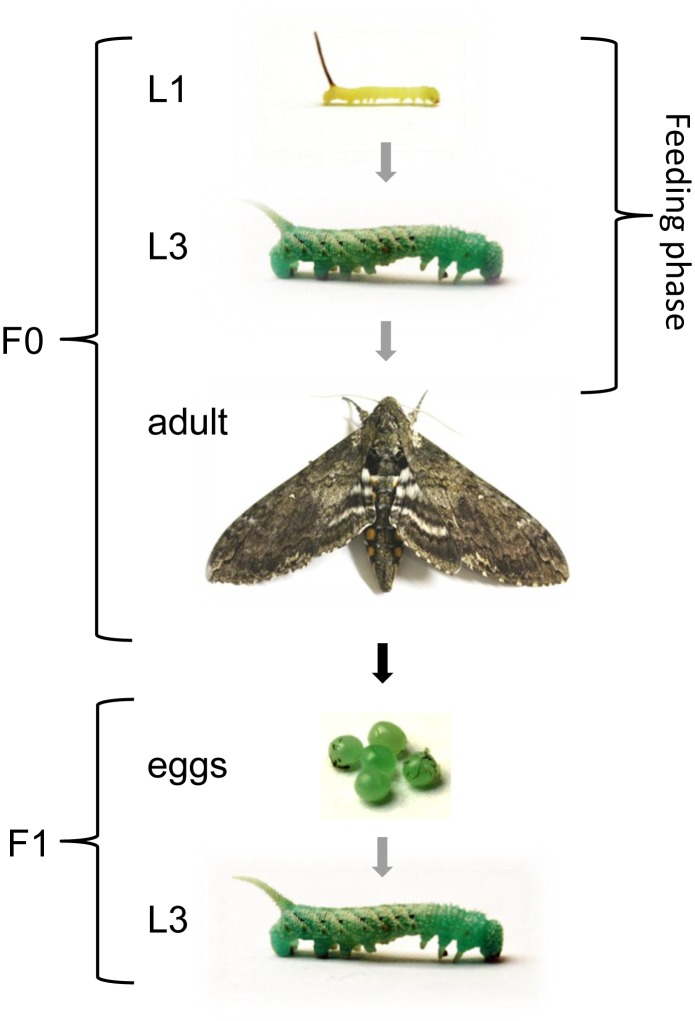
Experimental design to investigate TGIP in *M*. *sexta*. Larvae were fed on diets supplemented with *E*. *coli*, *S*. *entomophila* or fluorescence-labeled bacteria (Bioparticles) starting at the first instar and continuing until the first sampling point at the third instar. Except for the first instar, all displayed stages correspond to sampling points. Gray arrows indicate stage progression, the black arrow indicates generational progression, and the white double arrow indicates the duration of the feeding regimes. The life cycle proceeds as follows: five larval molts, one pupal molt, one imaginal molt, (embryonic) egg stage.

*Manduca sexta* eggs were collected from the in-house stock population for hatching and the larvae were maintained at 26°C, with 30% humidity and a 16-h photoperiod. We separated male larvae from female according to their dark spot in the posterior portion (Stewart et al., 1970). The larvae were reared on a standard artificial *M. sexta* diet (Bell and Joachim, 1976) drenched in overnight bacterial cultures of *E. coli* (9.5 × 10^7^ cfu/meal) or *S. entomophila* (1.5 × 10^8^ cfu/meal), or without bacteria as a control. Additionally, a group of larvae was reared on artificial diet drenched with 100 μl fluorescent BioParticles^®^ consisting of a mixture 1 mg/ml of chemically and heat-killed *E. coli* strain K-12 labeled with Texas Red^®^ (Molecular Probes) per 1 g of diet (Freitak et al., 2014). Larvae were fed *ad libitum* and food was replaced when needed or at least three times per week to ensure a steady supply of bacteria. Feeding was continued until the F0 larvae were removed for dissection at the third-instar stage, or throughout development in the case of specimens that were used to provide offspring for TGIP analysis. Parental development was monitored daily. After pupation, male and female specimens (2:1 ratio) were transferred to flight cages so they could begin mating after eclosion and wing maturation (one cage per treatment). Oviposited eggs were then counted daily for 10 consecutive days after initial oviposition on the provided substrates, i.e., tobacco plants (*Nicotiana tabacum*) and laboratory paper lining the cage walls. Eggs were allowed to hatch and F1 larvae were reared on an uncontaminated artificial diet until they were 1-day-old third-instars for the analysis of gene expression, histone H3 acetylation and DNA methylation (Figure 1). This experiment was repeated twice.

### Maternal Transfer of Bacteria

*Manduca sexta* third-instar larvae reared on a diet drenched with fluorescent BioParticles^®^ were embedded in Tissue-Tek^®^ OCT compound (Plano), flash-frozen in liquid nitrogen and stored at −80°C prior to cryostat sectioning. Abdomens, dissected ovaries and oviposited eggs from adult females were treated in a similar fashion. We prepared 10-μm cross-sections on a Leica CM 1850 Cryostat and viewed them under a Leica DM 5000 B fluorescence microscope with the N3 filter for Texas Red and the A4, L5 and Y5 filters as the negative control. Differential interference contrast (DIC) and bright field (BF) images were also captured to provide structural information. Fluorescent photomicrographs were acquired by overlaying the N3 and L5 filter cube images onto the DIC or BF images using the Leica LAS AF Lite image processing platform to optimize the fluorescence visualization.

### Identification of Sequences of Putative Epigenetic Regulatory Genes and Immunity-Related Genes

To identify putative *M*. *sexta* epigenetic regulators (e.g., HATs, HDACs and DNMTs), as well as immunity-related genes (e.g., encoding gloverin and lysozymes), we identified predicted and annotated *M*. *sexta* proteins based on the published genome sequence (Kanost et al., 2016). To confirm the annotated protein identities, the predicted amino acid sequences were used as queries for BLAST searches (using BLASTp with default parameters) against the NCBI nr database. Sequences with existing annotations matching the *M*. *sexta* sequences and with more than 55% amino acid sequence similarity to queries were collected for further analysis. All protein sequences were aligned in Geneious (vR10, Biomatters Ltd.) using MUSCLE with default settings, inspected for regions of high-quality alignment and refined manually. During this step, candidates were also scrutinized for the presence of conserved amino acid patterns.

### RNA Isolation and Quantitative Real-Time PCR

Midguts dissected from third-instar larvae (F0, F1) were homogenized in liquid nitrogen and total RNA was isolated using the PeqLab peqGOLD MicroSpin total RNA Kit. Sample quantity and purity were assessed using a NanoDrop spectrophotometer (PeqLab). If appropriate, samples were purified using RNeasy MinElute columns (Qiagen). First-strand cDNA was synthesized using the First Strand cDNA Synthesis Kit (Thermo Fisher Scientific) with 500 ng of DNA-free total RNA as the template and a 3:1 mixture of random hexamers and oligo-dT18 primers. Primers for real-time PCR were designed using Primer3 and available primer pairs were selected based on the lowest number of potential self-annealing structures and primer loops. Gene-specific primers are listed in Supplementary Table 1. The ribosomal protein L3 gene (*RPL3*) (Koenig et al., 2015) was used for normalization. Quantitative real-time PCR was conducted using an Applied Biosystems^®^ StepOnePlus^TM^ Real-Time PCR System on 96-well plates with the SensiMix^TM^ SYBR^®^ No-ROX Kit as the reporter mix. Each assay was repeated using three biological replicates (each representing pooled RNA from five third-instar larval midguts per sex) and two technical replicates. Fold changes in gene expression were calculated out using the 2^−ΔΔCt^ method (Livak and Schmittgen, 2001).

### Analysis of Histone Acetylation

Midguts were dissected from male and female third-instar larvae (F0, F1), flash frozen in liquid nitrogen, homogenized and stored at −80°C. Global levels of lysine-specific histone H3 acetylation were determined using the EpiQuik Global Histone H3 Acetylation Assay Kit (Epigentek Group Inc.) according to the manufacturer’s protocol. Fold changes of relative histone acetylation were calculated for treatment groups exposed to bacteria against the corresponding control groups.

### Preparation of DNA for Methylation Analysis

DNA was extracted from two replicates of five F0/F1 third-instar female/male larvae representing the control, *E*. *coli* and *S*. *entomophila* treatment groups using the GenElute^TM^ Mammalian Genomic DNA Miniprep Kit (Sigma-Aldrich). For each sample, the larvae were homogenized in liquid nitrogen and 150–200 μg of the resulting powder was used for DNA isolation, with a final elution volume of 100 μl. The DNA was precipitated by adding 10 μl 3 M sodium acetate (Carl Roth) and 200 μl ice-cold 100% (v/v) ethanol (Carl Roth), incubating at −20°C for at least for 2 h, and centrifuging at 4°C at 13,000 × *g* in a microfuge for 15 min (Sambrook and Russell, 2000). The pellet was washed with 20 μl ice-cold 70% (v/v) ethanol in Ambion nuclease-free water (Thermo Fisher Scientific) and dried at room temperature for 15 min before dissolving in 50 μl nuclease-free water on ice for 30 min. The DNA concentration was measured using a NanoDrop ND-1000 spectrophotometer (Thermo Fisher Scientific). If the A_260_/A_230_ ratio was less than 1.5, the DNA was purified using the NucleoSpin^®^ gDNA Clean-up kit (Macherey-Nagel) according to the manufacturer’s instructions.

### Global Analysis of DNA Methylation by LC-MS

DNA samples (1 or 2 μg) were digested with Degradase Plus (Zymo Research) at a ratio of 5 U/μg in a final volume of 25 μl overnight at 37°C, then diluted to 100 μl by adding 75 μl 0.1% (v/v) formic acid (ROTIPURAN^®^, Carl Roth) in ultrapure water (Milli-Q^®^ Advantage A10 water purification system, Merck Millipore) (Capuano et al., 2014). Calibration curves were prepared by dissolving 2′-deoxycytidine (dC, Sigma-Aldrich) and 5-methyl-2′-deoxycytidine (5mdC, Cayman Chemical) in nuclease-free water on ice, each to a final concentration of 1 mg/ml. The nucleoside stock solutions were diluted with 0.5% (v/v) formic acid in ultrapure water to yield 1, 2.5, 5, 10, 100, 250, 500, 1000, and 2000 pg/μl dC/^5*m*^dC-standard solutions. The analysis of genomic DNA was carried out by injecting 5-μl digested DNA samples and standard solutions into an UltiMate 3000 HPLC system (Dionex) followed by quantification in an amazon EDT ion trap mass spectrometer (Bruker Daltonics). Components were separated on a reversed-phase column (Kinetex C18, 2.6 μm, 50 × 2.1 mm, 100 Å, Phenomenex) under isocratic conditions [0.1% (v/v) formic acid (ROTIPURAN) and 5% (v/v) acetonitrile (ROTISOLV, Carl Roth) in ultrapure water] at a flow rate of 150 μl/min and 30°C. Cytidine residues were quantified by multiple reaction monitoring (MRM) after positive electrospray ionization using the following ion source parameters: 1.0 bar nebulizer pressure, 8 l/min drying gas, 200°C drying temperature, 4500 V capillary power and 500 V end-plate offset. Ionization and MRM conditions were optimized for fragmentation reactions for mass/charge ratios 228.1→112.0 (for dC) and 242.1→126.1 (for 5mdC). The data were analyzed using Compass Data Analysis v4.2 (Bruker Daltonics). Fold changes in relative global DNA methylation levels were calculated for treatment groups exposed to bacteria against the corresponding control groups.

### Statistical Analysis

We used the mean value for the parental and filial generations for every biological sample for males and females, as a control for natural cross-generational effects. We used the function *Summarize* of the R package FSA v0.8.17 (Ogle, 2016) to calculate means, medians, standard deviations, and standard errors of the mean for all experiments. The quantitative PCR results, global histone acetylation, and global DNA methylation data were analyzed for differences between sexes, treatments, and generations using R v3.2 as previously described (Gegner et al., 2018) (R script, Supplementary File 1). To test for differences in developmental times between treatment groups and controls, a Kruskal–Wallis multiple comparison test was applied with Bonferroni adjustment of *p*-values by using the function *dunnTest* in the package FSA v0.8.17.

## Results

To assess the potential molecular basis of TGIP in the parental generation, larvae were reared on a diet supplemented with microbes and we analyzed the expression of immunity-related genes as well as the levels of histone acetylation and DNA methylation. We also looked at developmental characteristics to determine the impact of TGIP on life history traits. In parallel, a group of larvae received a diet supplemented with fluorescent particles allowing us to visually monitor the uptake and fate of ingested microbes. In the F1 generation, the fate of the fluorescent particles was traced until the egg stage. In addition, gene expression, histone acetylation and DNA methylation were analyzed in the third larval instar (Figure 1).

### Diets Supplemented With Bacteria Affect Development

**FIGURE 2:**
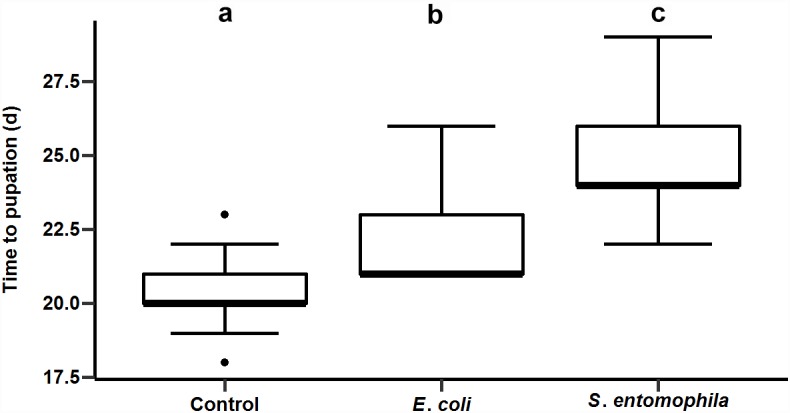
Impact of orally administered bacteria on larval development. The developmental time until pupation of 49 uninfected control larvae, 49 larvae infected with *E. coli*, and 41 larvae infected with *S. entomophila*. Boxplots show median, interquartile, and total regions. Significant differences between groups are depicted by different letters (a–c).

We supplemented larval diets with either the pathogen *S. entomophila* or the non-pathogenic bacterial species *E. coli* and monitored development compared to a control group fed on an uncontaminated diet. As shown before for other species, bacterial exposure delayed larval development significantly, i.e., by approximately 2 days in larvae exposed to *E*. *coli* and 4 days in larvae exposed to *S. entomophila* compared to untreated controls (*p* < 0.001). The larvae infected with pathogenic bacteria took 2 days longer to pupate than larvae exposed to the non-pathogenic bacteria (*p* < 0.001) (Figure 2 and Supplementary Tables 2, 3).

**FIGURE 3:**
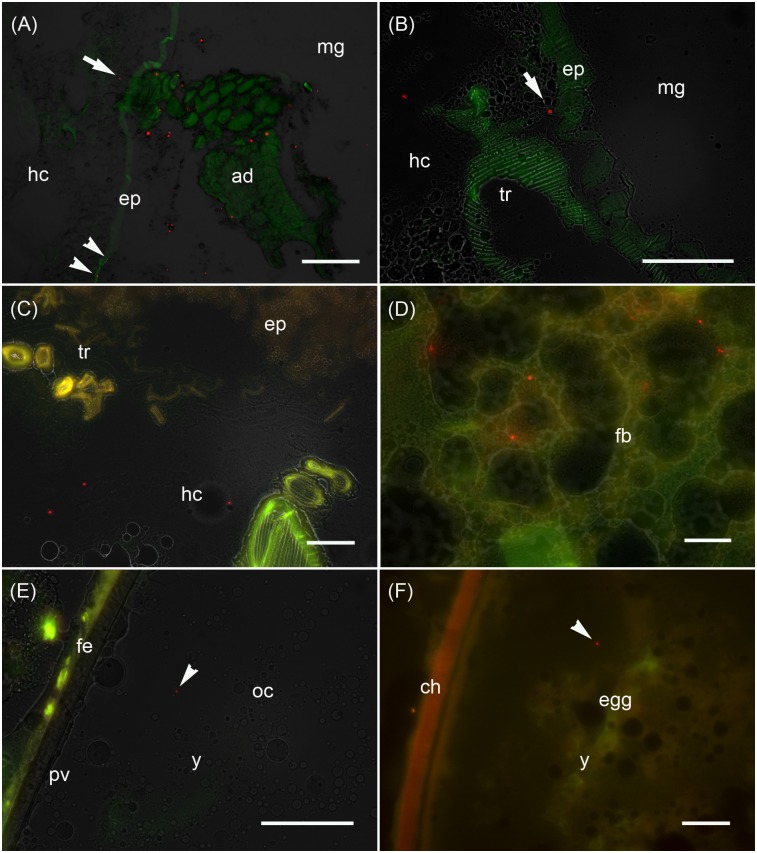
Analysis of the maternal transfer of bacteria in *M. sexta* by fluorescence microscopy Cryosection of third-instar larva **(A–D)**, adult ovary **(E)** and oviposited egg **(F)**. **(A)** Cross-sections of midgut region (md) after the uptake of artificial diet (ad) supplemented with *E. coli* Bioparticles labeled with Texas Red (red spots). Translocated Bioparticles are also detectable in the midgut epithelium (ep, arrowheads) and in the hemocoel (hc, arrow). Scale bar = 100 μm. **(B)** Magnification of the midgut region with adjacent tracheole (tr) shows Bioparticles immediately beneath the midgut epithelium (arrow). Scale bar = 100 μm. **(C)** Cross-sections of the midgut region reveal numerous Bioparticles distributed in the hemocoel. Scale bar = 50 μm. **(D)** Fluorescent bacteria are also abundant in the fat body tissue (fb). Scale bar = 50 μm. **(E)** Female reproductive system dissected from adults reared on artificial diet containing Bioparticles during earlier larval stages. Fluorescent bacteria (arrowhead) are located in the yolk mass (y) of a developing oocyte (oc) which is surrounded by a thin perivitelline membrane (pv) and the follicle epithelium (fe). Scale bar = 100 μm. **(F)** Ovipositioned egg (egg) with an enclosing chorion (ch) contains bacterial probes (arrowhead) in the yolk mass (y). Scale bar = 50 μm.

### Bacteria Can Be Transferred From Mothers to Offspring

We monitored the transfer of bacteria from mothers to their offspring using non-viable *E. coli* labeled with the fluorescent dye Texas Red. These bacteria were added to the larval diet and visualized by fluorescence microscopy in cryosections of third-instar F0 larvae (Figure 3A–D), ovaries of adult females derived from these larvae (Figure 3E), and oviposited F1 eggs (Figure 3F). Using this approach, we determined that the labeled bacteria can translocate from the midgut lumen into the hemocoel, where they attach to the fat body (Figure 3). The translocated bacteria are then deposited in the ovaries and taken up into the developing eggs. The labeled bacteria were associated with the follicle epithelium, the ovariole wall and the vitelline membrane. The translocated gut-derived bacteria were ultimately detected in the laid F1 eggs among yolk proteins and lipids (Figure 3).

### TGIP Affects the Expression of Several Immunity-Related Genes in a Sex-Specific Manner

**FIGURE 4:**
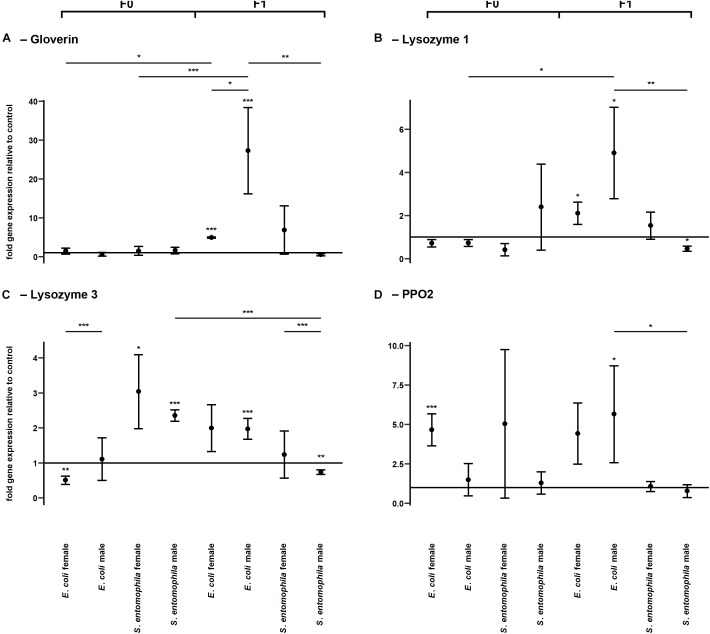
Differential expression of immunity-related genes. Relative expression levels of immunity-related genes in the parental generation reared on bacterial diets (pathogenic and non-pathogenic) or uncontaminated control diets (F0) and unchallenged offspring (F1). The target genes were: **(A)** gloverin, **(B)** lysozyme 1, **(C)** lysozyme 3, and **(D)** pro-phenoloxidase 2. Relative fold changes for each gene were set to 1 for the control treatment and normalized against the 60S RNA housekeeping gene. Results represent the mean values of three independent biological replicates and error bars indicate the standard error of the mean. Significance levels: ^∗^*p* < 0.05, ^∗∗^*p* < 0.01, ^∗∗∗^*p* < 0.001.

To determine whether TGIP influences the expression of immunity-related effector genes, namely those encoding gloverin, lysozyme isoforms 1 and 3, and pro-phenoloxidase 2 (PPO2), F0 third-instar larvae were fed on diets supplemented with either *S. entomophila* or *E. coli* (Figure 4 and Supplementary Table 4). In both groups challenged with bacteria, gloverin gene expression was unaffected in the parental generation (Figure 4A). However, the lysozyme 3 gene was significantly upregulated in fathers (*p* < 0.001) and mothers (*p* < 0.05) fed on diets supplemented with *S. entomophila*, but downregulated in mothers fed on diets supplemented with *E. coli* (*p* < 0.01) (Figure 4C). In the latter group, *PPO2* was also significantly upregulated (*p* < 0.001) (Figure 4D). Remarkably, we observed sex-specific changes in gloverin expression (*p* < 0.05) in third-instar F1 larvae from parents challenged with *E. coli*, whereas there was no sex-specific difference in third-instar F1 larvae from parents challenged with *S. entomophila* or in larvae from parents fed on the uncontaminated control diet. Gloverin was upregulated in both sexes (*p* < 0.001) but this was more pronounced in males (*p* < 0.05). Both lysozyme isoforms were also specifically upregulated in F1 males (lysozyme 1, *p* < 0.05; lysozyme 3, *p* < 0.001) (Figure 4B,C) and so was *PPO2* (*p* < 0.05). For all of these transcripts, male larvae originating from parents challenged with *E. coli* showed stronger upregulation than males stemming from parents challenged with *S. entomophila* (gloverin, *p* < 0.01; lysozyme 1, *p* < 0.01; lysozyme 3, *p* < 0.001; PPO2, *p* < 0.01). The latter group either showed no differences compared to the control cohort or the genes were slightly downregulated (lysozyme 1, *p* < 0.05; lysozyme 3, *p* < 0.01). In female offspring of parents challenged with *E. coli*, gloverin and lysozyme 1 were significantly upregulated compared to the control cohort (gloverin, *p* < 0.001; lysozyme 1, *p* < 0.05) but the other genes were not. Furthermore, there was no difference in expression between female offspring of parents exposed to the two different species of bacteria.

### TGIP Also Influences Histone Acetylation/Deacetylation

**FIGURE 5:**
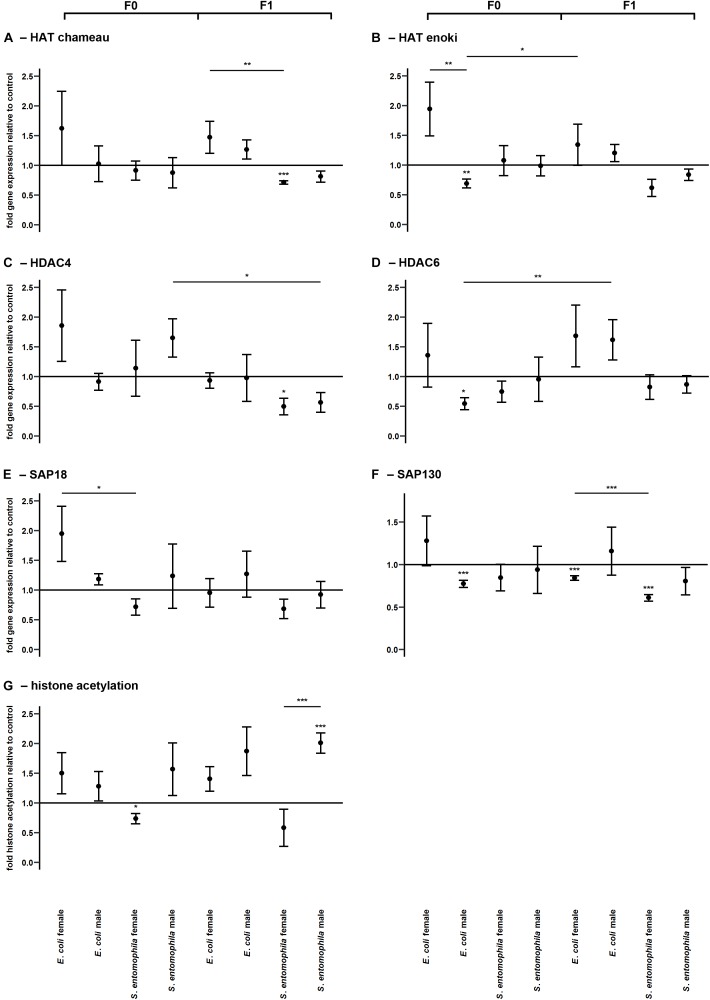
Differential expression of genes encoding histone modifying enzymes and corresponding levels of histone acetylation. Relative expression levels of genes associated with histone modification in the parental generation reared on bacterial diets (pathogenic and non-pathogenic) or uncontaminated control diets (F0) and unchallenged offspring (F1). The target genes were: **(A)** HAT chameau, **(B)** HAT enoki, **(C)** HDAC4, **(D)** HDAC6, **(E)** SAP18, and **(F)** SAP130. **(G)** Corresponding levels of relative histone H3 acetylation. Relative fold changes for each gene were set to 1 for the control treatment and normalized against the RPL3 housekeeping gene. Results represent the mean values of three independent biological replicates for relative gene expression and four replicates for relative histone acetylation. Error bars indicate the standard error of the mean. Significance levels: ^∗^*p* < 0.05, ^∗∗^*p* < 0.01, ^∗∗∗^*p* < 0.001.

The potential epigenetic basis of TGIP was investigated by monitoring the larvae fed on contaminated and uncontaminated diets for the expression profiles of representative genes encoding either HATs (HAT enoki and HAT chameau) or HDACs (HDAC4, HDAC6, SAP18, and SAP130), and comparing the expression profiles of the same genes in the F1 larvae (Figure 5 and Supplementary Table 6). Accordingly, we found that HAT enoki was upregulated in fathers challenged with *E. coli* (*p* < 0.01) and HAT chameau was downregulated in female offspring of parents challenged with *S. entomophila* (*p* < 0.001) (Figure 5A,B). For SAP18, we detected a significant treatment-specific difference between the mothers in the different treatment groups, with higher expression in the *E. coli* group (*p* < 0.05) (Figure 5E). Fathers challenged with *E. coli* displayed a reduced capacity for deacetylation. We observed the significant downregulation of HDAC6 (*p* < 0.05) and SAP130 (*p* < 0.001) (Figure 5D,F). In the F1 generation, female offspring of parents challenged with *S. entomophila* showed a reduced capacity for deacetylation, with a significant downregulation of HDAC4 (*p* < 0.05) and SAP130 (*p* < 0.001) (Figure 5C,F). SAP130 was also significantly downregulated in female larvae whose parents were exposed to *E. coli* (*p* < 0.001), but there was a less significant reduction compared with female larvae whose parents were exposed to *S. entomophila* (*p* < 0.001).

Surprisingly, these differences in HATs and HDACs were only partially reflected by the actual relative histone acetylation levels. Mothers exposed to *S. entomophila* displayed a slight but significant reduction in global histone H3 acetylation compared to the control (*p* < 0.05). Male F1 larvae from these mothers displayed significantly higher levels histone H3 acetylation than their counterparts whose parents were not exposed to bacteria (*p* < 0.001) and also when compared to their female peers (*p* < 0.001) (Figure 5G and Supplementary Table 6).

### Impact of TGIP on DNA Methylation

**FIGURE 6:**
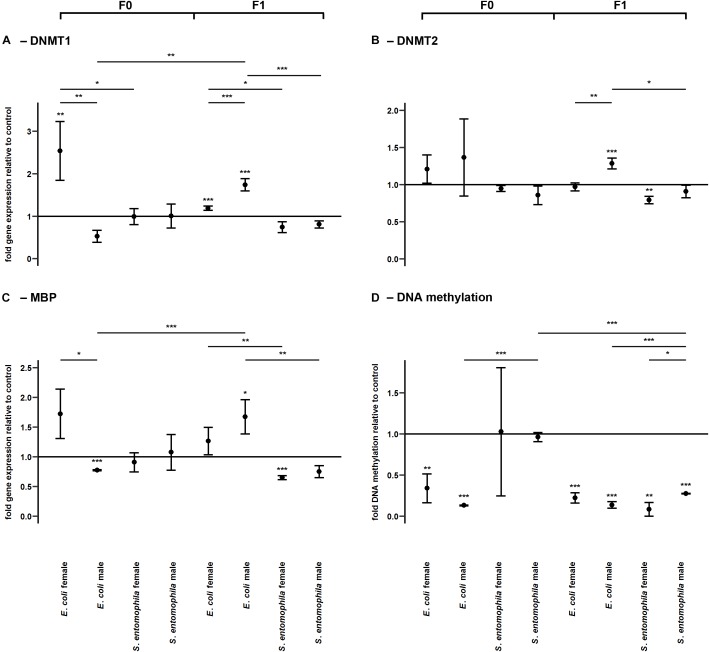
Differential expression of genes encoding DNA modifying enzymes and corresponding levels of DNA methylation. Relative expression levels of genes associated with DNA methylation in the parental generation reared on bacterial diets (pathogenic and non-pathogenic) or uncontaminated control diets (F0) and unchallenged offspring (F1). The target genes were: **(A)** DNMT1, **(B)** DNMT2, and **(C)** MBD. **(D)** Corresponding levels of relative global DNA methylation. Relative fold changes for each gene were set to 1 for the control treatment and normalized against the *RPL3* housekeeping gene. Results represent the mean values of three independent biological replicates for relative gene expression and two replicates for the relative DNA methylation. Error bars indicate the standard error of the mean. Significance levels: ^∗^*p* < 0.05, ^∗∗^*p* < 0.01, ^∗∗∗^*p* < 0.001.

DNA methylation was assessed by monitoring the larvae fed on contaminated and uncontaminated diets for the expression profiles of representative genes encoding DNMT1, DNMT2, and MBD (Figure 6 and Supplementary Table 9). The expression profiles of the same genes were determined in F1 larvae for comparison. There was a sex-specific difference for both *DNMT1* (*p* < 0.01) and *MBD* (*p* < 0.05) expression in the *E. coli* group. These genes were significantly upregulated in mothers compared to fathers (Figure 6A,C). Interestingly, *DNMT1* also was significantly upregulated in mothers exposed to *E. coli* relative to those challenged with *S. entomophila* (*p* < 0.05). There also was a sex-specific difference in the expression of *DNMT1* (*p* < 0.001) and *DNMT2* (*p* < 0.01) in F1 larvae of parents challenged with *E. coli*. These enzymes were significantly more upregulated in males than in females, and also with respect to F1 male larvae from mothers challenged with *S. entomophila* (*DNMT1*, *p* < 0.001; *DNMT2*, *p* < 0.05) (Figure 6A,B). This treatment-specific difference in gene expression was also observed for MBD across both sexes (males, *p* < 0.01; females, *p* < 0.01).

The relative global DNA methylation levels ranged between 0.09 ± 0.13% and 1.26 ± 0.08% (Supplementary Table 11). For the *E. coli* treatment group, we observed significantly reduced levels of DNA methylation in both sexes (males, *p* < 0.001; females, *p* < 0.01) but no such effect was found in the *S. entomophila* treatment group. Interestingly, DNA methylation also was significantly lower in fathers challenged exposed to *E. coli* compared to those treated with *S. entomophila* (*p* < 0.001). On the other hand, in the F1 generation, male and female offspring of both parents in both treatment groups displayed significantly reduced DNA methylation levels compared to controls (*E. coli* group males and females, *p* < 0.001; *S. entomophila* group males, *p* < 0.001; *S. entomophila* group females, *p* < 0.01). In larvae stemming from parents challenged with *S. entomophila* there also was a sex-specific difference (*p* < 0.05), with females displaying significantly lower methylation levels than males. Additionally, the latter showed significantly higher DNA methylation levels than the male offspring of parents challenged with *E. coli* (*p* < 0.001) (Figure 6D and Supplementary Table 9).

## Discussion

Theory predicts that immune responses will be sex-specific because the reproductive effort of females is higher than that of males. According to Bateman’s principle, males improve their fitness by increasing their mating success whereas females increase fitness through longevity (Rolff, 2002). Current evidence suggests that these different investment strategies and life-history traits translate into the sex-specific expression of immunity-related genes in insects, which is in turn reflected by the sex-specific expression of regulatory microRNAs (Jacobs et al., 2016). Several studies have shown that parental investment into their offspring is achieved via TGIP which is also sex-dependent (Roth et al., 2010; Eggert et al., 2014). The higher specificity of maternal TGIP in insects can be explained by – but may not be limited to – the transfer of specific bacterial or fungal cells from the mother to the offspring (Freitak et al., 2014; Fisher and Hajek, 2015). However, it is unclear how male insects can transmit information about the pathogens they have encountered, and we have previously hypothesized that epigenetic mechanisms could explain this phenomenon (Vilcinskas, 2016; Vilcinskas, 2017). We selected the tobacco hornworm (*M. sexta*) because it is a widely used model of insect physiology and immunity and previous studies have demonstrated that TGIP occurs in this species (Trauer and Hilker, 2013; Trauer-Kizilelma and Hilker, 2015a,b; Rosengaus et al., 2017). Although speculating on the potential epigenetic dimension of their findings, these earlier studies did not address this topic by way of design. To the best of our knowledge the work of Castro-Vargas et al. (2017) is the only study which investigated the influence of an epigenetic regulator in TGIP. In this work the researchers found that RNA methylation is related to immune priming within but not across generations in *T*. *molitor* while DNA methylation was not detected (Castro-Vargas et al., 2017). As histone acetylation assays were previously established in our group for *G*. *mellonella* (Mukherjee et al., 2012), and DNA methylation was detected in closely related species, for example *B*. *mori* and *M*. *brassica* (Bewick et al., 2017), these two epigenetic mechnisms provided a promissing system to investigate the potential link between epigenetics and TGIP in our model organism *M*. *sexta*.

Given that pathogenic and non-pathogenic bacteria can influence TGIP in different ways, we fed hornworm larvae on diets supplemented with either the entomopathogenic species *S. entomophila* or the common gut inhabitant *E. coli* to mimic natural oral infections. The supplemented diets confirmed that *S. entomophila* and *E. coli* cause different developmental effects. Similar findings in *G. mellonella* have been attributed to the ability of pathogens, but not non-pathogenic species, to interfere with epigenetic mechanisms in the infected host (Mukherjee et al., 2015; Vilcinskas, 2017).

Next, we demonstrated that bacteria added to the diet of female larvae can translocate from the gut into the hemocoel and are ultimately deposited in the eggs, where they have the potential to elicit an immune response that may be sufficient to protect offspring from pathogens as they hatch (Figure 3). We also found that orally delivered *E. coli* and *S. entomophila* modulated the expression of selected immunity-related genes (encoding gloverin, two lysozyme isoforms, and a pro-phenoloxidase) in the gut of the infected F0 larvae, and also in their offspring. Interestingly, we observed significant sex-specific differences in the expression of gloverin in third-instar F1 larvae whose parents were exposed to *E. coli*, providing evidence for sex-specific TGIP in *M. sexta*. The near universal pattern was that male offspring of parents challenged with *E. coli* displayed the highest expression levels of immunity-related genes among all tested subgroups (Figure 4A,C,D). This agrees with an earlier report showing that *M. sexta* gloverin was induced to significantly higher levels in the hemocytes and midgut by Gram-negative *E. coli* than by other microorganisms (Xu et al., 2012). Male larvae therefore express antimicrobial peptides at higher levels than females, but the reason for this is unclear because the phenomenon does not seem to fit Bateman’s principle. However, the activation of innate immunity constitutes fitness costs as well as benefits, so organisms are expected to optimize (but not necessarily maximize) their immune responses according to the circumstances. Optimality models predict that when pathogens are particularly detrimental to male mating success but have a less severe effect on female fecundity or longevity, a superior male immune response will evolve (Stoehr and Kokko, 2006). For example, freshly eclosed *Pieris rapae* adult males display a stronger encapsulation response than females, but the female response becomes stronger as the individuals age (Stoehr, 2007). In our study, we analyzed gene expression only during the third larval stage. It is possible that the immune system dynamics are switched in favor of females during later larval stages, the pupal stage or at some point during the adult phase. However, for males to give preference to reproductive success over immunity, they would need to become sexually mature in the first place. If they succumb to pathogens during the larval stage, this trade-off becomes irrelevant. For example, in the case of *PPO2* (which is needed for the encapsulation response) we detected a near one-to-one transmission of the expression profile from parents challenged with *E. coli* to their male offspring (Figure 4D). Perhaps mothers specifically provide their male offspring with a form of immune competence that they would otherwise not exhibit due to a built-in trade-off. Moreover, in contrast to the results of an earlier investigation of transgenerational immune gene expression in *M. sexta* (Trauer-Kizilelma and Hilker, 2015b), we detected induced gloverin expression even though offspring remained unchallenged. This may reflect the different routes of infection and the different priming agents used in each study, and the different offspring stages assessed to investigate TGIP. The authors of this earlier study injected their parental generation with a peptidoglycan isolated from the Gram-positive bacterium *Micrococcus luteus* and sampled F1 eggs and ovaries of F1 females (Trauer-Kizilelma and Hilker, 2015b), whereas we used oral infection with Gram-negative *E. coli* and sampled male and female third-instar larvae. Similar findings to those reported herein were presented following the oral infection of *G. mellonella* with a mixture of Gram-negative *E. coli* and Gram-positive *M. luteus*, or with the entomopathogenic species *Pseudomonas entomophila* or *S. entomophila* (Freitak et al., 2014). The gloverin gene was upregulated in the eggs of challenged females and gloverin levels in their *E. coli*/+*M. luteus* challenge group were similar to the levels we observed in *M. sexta*.

Interestingly the bona fide pathogen we tested (*S. entomophila*) did not elicit any transgenerational immune responses, regardless of the offspring sex. If anything, we observed a slight downregulation of transcripts encoding immune effectors in this group (Figure 4). This may reflect the pathogen-induced circumvention of host efforts to protect their offspring against previously encountered microbes. If true, this would be consistent with the classical paradigm of host–parasite co-evolution and could represent a case of reciprocal epigenetic adaptations, as previously suggested (Vilcinskas, 2016). Accordingly, in the offspring generation, we found that the expression of HAT chameau was significantly downregulated in females whose parents had been exposed to *S. entomophila*, whereas the same gene was upregulated in females whose parents had been exposed to *E*. *coli*. We did not observe any differential regulation of HAT enoki in the *S*. *entomophila* treatment group but sex-specific regulation was evident in the *E. coli* treatment group (elevated in mothers but repressed in fathers) indicating that histone acetylation may underlie the sex-specific TGIP we observed (Figure 5A,B). Our interpretation relies on the general notion that the acetylation of histones H3 and H4 is highly correlated with gene expression, which seems to be conserved across higher eukaryotes (Zhang et al., 2015). At the same time, the expression of the HDAC SAP18 was significantly elevated in *E. coli*-fed mothers, which indicates ongoing differential gene regulation across the genome, consistent with adaptive processes that might contribute to TGIP. In line with this interpretation, HDAC6 and SAP130 were significantly downregulated in *E. coli*-fed fathers. Interestingly, the relative levels of histone H3 acetylation were lower only in the mothers challenged with *S. entomophila*, which again indicates pathogen-derived epigenetic interference that results in overall transcriptional repression (Figure 5G). This agrees with an earlier study showing that pathogenic bacteria can interfere with the regulation of HDACs and HATs in insects and can manipulate host immunity in *G. mellonella* (Mukherjee et al., 2012).

In the F1 offspring of parents challenged with *S. entomophila*, HDAC4 and SAP130 were significantly downregulated in female F1 larvae, which surprisingly did not correlate with the higher relative histone H3 acetylation levels. On the other hand, there was no differential regulation of histone acetylation modifiers in F1 male larvae, but the level of histone H3 acetylation was significantly higher. These results may indicate that the causal relationship between histone modifiers and marks is not as straightforward in lepidopteran species as previously assumed. Perhaps crosstalk with other histone marks such as methylation and ubiquitinylation have a dominant regulatory effect over the state of histone acetylation than the enzymes responsible for the actual addition and removal of acetyl groups. Alternatively, these results may represent prolonged sex-specific interference by the entomopathogen to disrupt the epigenetic machinery of the host well into the larval stage of the next generation, perhaps making males more susceptible to attack through the deregulation of immunity-related gene expression. Given that histone modifications occur locally due to the DNA sequence-dependent binding of transcription factors that recruit the HATs and HDACs (Zhang et al., 2015), the abundance of specific enzymes does not necessarily mean that global acetylation levels must change in the same manner.

In addition to histone acetylation, we also observed a sex-specific difference in the expression of genes related to DNA methylation when the parents were exposed to *E. coli*. DNMT1 and MBD were significantly upregulated in mothers but significantly downregulated in fathers (Figure 6A,C). Surprisingly, global DNA methylation levels were reduced in parents fed on diets containing *E. coli* but there was no significant difference between sexes. It is unclear why upregulation of the maintenance methyltransferase DNMT1 did not prevent the observed demethylation in *E. coli*-fed mothers. DNMT1 may perform additional functions other than maintaining the DNA methylation status across cell cycles in *M. sexta*, or the oral challenge with *E. coli* may have triggered the major reprogramming of the methylome. It is conceivable that new genomic regions were methylated *de novo* while a larger, previously methylated portion of the genome became demethylated as a response to the infection, explaining the (partially sex-specific) gene expression we observed in their offspring. Ten-eleven translocation dioxygenases have been implicated in active DNA demethylation in vertebrates, but the demethylation apparatus in insects is unknown (Provataris et al., 2018).

Interestingly, in parents exposed to *S. entomophila*, there was no change in the expression of methylation-related enzymes or in the global DNA methylation status (Figure 6D). This further supports our hypothesis that the pathogen was able to interfere with or even subdue the epigenetic machinery of the host, as has recently been demonstrated in the diamondback moth (*Plutella xylostella*) during infections with the koinobiotic endoparasitic wasp *Cotesia plutellae* (Kumar and Kim, 2017). DNA methylation was reduced in parasitized larvae relative to non-parasitized controls, especially at late parasitic stages, along with reduced expression levels of DNMT1, DNMT2 and MBD. The mechanisms of epigenetic interference used by parasitic wasps and pathogenic bacteria are likely to differ substantially, given the phylogenetic distance between the invaders. Infection with *S. entomophila* did not alter DNA methylation in the parental generation but methylation levels were lower in the F1 offspring, particularly in females. Furthermore, and as observed in *P. xylostella*, the expression of DNMT1, DNMT2 and MBD in F1 larvae of both sexes was much lower when the parents had been challenged with *S. entomophila* compared to *E. coli* (Figure 6A–C). *S. entomophila* therefore appears to suppress TGIP by interfering with gene expression in the offspring. In contrast, in the female offspring of *E. coli*-fed parents there was no change or limited upregulation of the enzymes involved in DNA methylation whereas there was consistent upregulation in males, as observed for the expression of immunity-related genes. In both sexes, DNA methylation levels were lower than in the control treatment group. This configuration of enzymes versus methylation status again points toward an active and ongoing restructuring of epigenetically mediated gene regulation. Even though at first glance the reduced DNA methylation in both offspring groups seems to be analogous, this evidently does not translate into similar transcriptional profiles, as explained above. Interestingly, in the cotton bollworm *Helicoverpa armigera*, DNA methylation is tightly associated with stably expressed genes with basic housekeeping roles, such as transcription and translation (Jones et al., 2018). Even minute differences in such basic but diverse biological functions are likely to result in profoundly different transcriptional outcomes, depending on the methylation state of the corresponding genomic region.

## Conclusion

We have shown that TGIP in *M. sexta* is associated with changes in both histone acetylation and DNA methylation. Effects in the offspring depended on the species of bacteria encountered by the parents, and were sex-specific for certain genes as well as for histone acetylation. We have also demonstrated that infection with non-pathogenic *E. coli* resulted in the differential expression of immunity-related genes and DNA methylation-modifying enzymes in the offspring generation, with the highest expression levels observed in males. The entomopathogen *S. entomophila* appears to influence most of the parameters we tested, consistent with counteracting the TGIP efforts of the host. The latter hypothesis warrants more research to determine the extent to which the observed effects reflect an epigenetic dimension of host–parasite coevolution. Our study shows that epigenetic mechanisms are promising tools to get further insight in the molecular mechanisms behind TGIP.

## Data Availability

The datasets for this study can be found in the Supplementary Material.

## Author Contributions

AB and JG carried out the laboratory work related to the TGIP experiments. JG contributed to data analysis. RH and JG analyzed DNA methylation. KM analyzed histone acetylation. HV designed primers for qPCR and identification of epigenetic markers and immune genes. AV designed the study, provided funding, and supervised AB and JG. All authors drafted parts of the manuscript, gave approval for publication and agree to be accountable for the content.

## Conflict of Interest Statement

The authors declare that the research was conducted in the absence of any commercial or financial relationships that could be construed as a potential conflict of interest.
