# Supplementary material for: Epigenetic Mechanisms Are Involved in Sex-Specific Trans-Generational Immune Priming in the Lepidopteran Model Host Manduca sexta
Source: Front Physiol. 2019 Mar 4;10:137. doi: 10.3389/fphys.2019.00137 (PMC6410660; doi:10.3389/fphys.2019.00137)
Supplement: Supplementary file 1 [file Data_Sheet_1.pdf]

R script for statistical analysis of qPCR data, relative histone acetylation, and relative DNA methylation

```
library(multcomp)
library(FSA)
library(dplyr)
set.seed(12345)

# Input for analysis of ddCT of immune genes and epigenetic regulatory genes, and of relative histone acetylation and relative DNA methylation
data <- read.delim("~/Input.txt", header = T)

#calculation of mean, standard deviation and standard error
data2 <- Summarize(X~gts, data = data)
data2$se <- data2$sd/sqrt(data2$n)
#one-way ANOVA for generation vs treatment vs sex
a <- aov(X~gst, data = data)

#test of normalized data: t-quantile-quantile-plots
qqt.sim <- function(m) {
  oldp <- par(mfrow = c(3, 4), mar = c(3, 3.5, 2, 0.1),
              mgp = c(1.5, 0.5, 0), tcl = -0.3)
  stdres <- rstandard(m)
  n <- length(stdres)
  df <- m$df.residual
  qqplot(x = qt(ppoints(n), df = df), y = stdres, col = "red",
         ylab = "Empirical quantiles of\nstandardized residuals",
         xlab = paste("t-quantiles with", df, "d.o.f."),
         main = "Model residuals")
  qqline(y = stdres, distribution = function(p) qt(p, df = df))
  replicate(11, {
    xx <- rt(n, df = df) #n t-distr. data with same df as empirical data
    qqplot(x = qt(ppoints(n), df = df), y = xx,
           ylab = "Empirical quantiles",
           xlab = paste("t-quantiles with", df, "d.o.f."),
           main = "Simulated t-data")
    qqline(y = xx, distribution = function(p) qt(p, df = df))
  })
  invisible(par(oldp))
}
```

```
qqt.sim(m)

#post-hoc analysis
ph <- glht(a, linfct = mcp(gst = c("Cf-F0Ef=0","Cf-F0Sf=0","Cf-F1Ef=0","Cf-F1Sf=0","Cm-F0Em=0",
                                    "Cm-F0Sm=0","Cm-F1Em=0","Cm-F1Sm=0","F0Ef-F0Em=0",
                                    "F0Ef-F0Sf=0","F0Ef-F1Ef=0","F0Em-F0Sm=0","F0Em-F1Em=0",
                                    "F0Sf-F0Sm=0","F0Sf-F1Sf=0","F0Sm-F1Sm=0","F1Ef-F1Em=0",
                                    "F1Ef-F1Sf=0","F1Em-F1Sm=0","F1Sf-F1Sm=0")), vcov = sandwich)
```

#Abbreviations: X = ddCT; relative histone acetylation, or relative DNA methylation;
gts = generation:treatment:sex;
F0 = parental generation;
F1 = first filial generation;
C = control;
E = *E. coli*;
S = *S. entomophila*;
f = female;
m = male
