# Supplementary material for: Epigenetic Mechanisms Are Involved in Sex-Specific Trans-Generational Immune Priming in the Lepidopteran Model Host Manduca sexta
Source: Front Physiol. 2019 Mar 4;10:137. doi: 10.3389/fphys.2019.00137 (PMC6410660; doi:10.3389/fphys.2019.00137)
Supplement: Supplementary file 2 [file Data_Sheet_2.docx]

Supplementary Material

Epigenetic mechanisms mediate sex-specific trans-generational immune priming in the lepidopteran model host *Manduca sexta*

Jasmin Gegner^1§^, Arne Baudach^2§^, Krishnendu Mukherjee^2^, Rayko Halitschke^3,4^, Heiko Vogel^3^, Andreas Vilcinskas^1,2*^

*** Correspondence:** Andreas Vilcinskas: [Andreas.Vilcinskas@agrar.uni-giessen.de](mailto:Andreas.Vilcinskas@agrar.uni-giessen.de)

# Supplementary Files

**Supplementary File 1.** R script for statistical analysis of qPCR data, relative histone acetylation, and relative DNA methylation.

see separate PDF file ‘Supplementary File1’

# Supplementary Figures Tables

**Supplementary Table 1.** *M. sexta* primer sequences for immune genes and epigenetic modifiers.

| **Transcript** | **Forward Primer** | **Reverse Primer** |
| --- | --- | --- |
| **Gloverin** | CTGACTGCCAGTGGATCAGG | ACCTTGGAAGCCGACATCAG |
| **Lysozyme1** | GCGAGGCTAAACACTTCAGC | TCATTCTCCACGAGGCACAC |
| **Lysozyme3** | TCGGTGCAAGGGACAAGTAC | TTGCCATTCGAGGTCTGGAC |
| **PPO2** | GCAGCAGTCATCCCGAAAAC | CGAGATGCCAATGCCAATGG |
| **HAT chameau** | TGGCTAAAGGCAGGACTACTC | GGGGACTTGCTCTCTGAATC |
| **HAT enoki** | ACCCTCCAAACAGAAGAAGC | TGGGAGCAGCATATCAGTG |
| **HDAC4** | CCAAGAAACAACCATATCGTG | CCCACAGTTTCAAATGTTCC |
| **HDAC6** | CGGCTACTGCTTCTACAACAAC | TGGGTCATCGTAAAACATCTG |
| **SAP18** | CGGTTGATAGGGAAAAGACTTG | TGTTCCTTTACGCCTAGTTTCAG |
| **SAP130** | CCAGTGGGCAAATAATCAGTC | GTTTGGCAGTTGACAGCATTAC |
| **DNMT1** | CAAGACGCTGTGGAGGAATAC | CACAGATGCCCTTGTTCATC |
| **DNMT2** | GCAACATTCAAGCACTTTCAC | CAGTCCTTGAGTCATTTTCGTC |
| **MBD** | GCGATTGGAAGGTTTAACAGC | TTGCGACGAAACATGAAGAG |

**Supplementary Table 2.** Results of statistical analysis for developmental time till pupation of larvae fed with *E*. *coli*- or *S*. *entomophila*-containing diet or standard diet (control). A Kruskal-Wallis multiple comparison p-values adjusted with the Bonferroni method was performed. Significance levels: p < 0.05 (*), p < 0.01 (**), p < 0.001 (***). The corresponding raw data can be found in Supplementary Table 3.

|  | **Z** | **P value, unadjusted** | **P value, adjusted** | **Significance level** |
| --- | --- | --- | --- | --- |
| **Control against *E*. *coli*** | -4.895 | 9.842$\cdot$10^-07^ | 2.952$\cdot$10^-06^ | ******* |
| **Control against *S*. *entomophila*** | -9.567 | 1.097$\cdot$10^-21^ | 3.292$\cdot$10^-21^ | ******* |
| ***E*. *coli* against *S*. *entomophila*** | -4.895 | 9.824$\cdot$10^-07^ | 2.947$\cdot$10^-06^ | ******* |

**Supplementary Table 3.** **(A)** Developmental times till pupation and of larvae fed with *E*. *coli*- or *S*. *entomophila*-containing diet or standard diet (control), and **(B)** their corresponding mean, median, standard deviation (Sd), and standard error of the mean (Se).

see separate Excel file ‘Supplementary Table3’

**Supplementary Table 4.** Statistical analysis for ΔΔC_T_ data of immune genes. A parametric multiple comparisons analysis utilizing simultaneous tests for general linear hypotheses on a fitted one way ANOVA model was performed and p-values were adjusted using sandwich estimator. Results for *E*. *coli*- (E) and *S*. *entomophila*- (S) infected females (f) and males (m) of the parental (F0) and filial (F1) generation compared to control (C) females and males. Significance levels: p < 0.05 (*), p < 0.01 (**), p < 0.001 (***).The corresponding raw data can be found in Supplementary Table 5.

|  | **Linear hypotheses** | **Estimate** | **Standard error** | **F value** | **P value** | **Significance level** |
| --- | --- | --- | --- | --- | --- | --- |
| **Gloverin** | Cf-F0Ef==0 | -0.070 | 0.688 | -0.102 | 1.000 |  |
|  | Cf-F0Sf==0 | 0.365 | 0.943 | 0.387 | 1.000 |  |
|  | Cf-F1Ef==0 | -2.304 | 0.038 | -61.140 | <0.001 | ******* |
|  | Cf-F1Sf==0 | -0.825 | 1.487 | -0.555 | 0.999 |  |
|  | Cm-F0Em==0 | 1.762 | 1.005 | 1.753 | 0.594 |  |
|  | Cm-F0Sm==0 | 0.298 | 1.234 | 0.242 | 1.000 |  |
|  | Cm-F1Em==0 | -4.458 | 0.584 | -7.636 | <0.001 | ******* |
|  | Cm-F1Sm==0 | 1.952 | 1.171 | 1.666 | 0.650 |  |
|  | F0Ef-F0Em==0 | 1.832 | 1.218 | 1.504 | 0.753 |  |
|  | F0Ef-F0Sf==0 | 0.435 | 1.167 | 0.373 | 1.000 |  |
|  | F0Ef-F1Ef==0 | -2.234 | 0.689 | -3.241 | 0.046 | ***** |
|  | F0Em-F0Sm==0 | -1.464 | 1.591 | -0.920 | 0.977 |  |
|  | F0Em-F1Em==0 | -6.220 | 1.162 | -5.351 | <0.001 | ******* |
|  | F0Sf-F0Sm==0 | -0.067 | 1.553 | -0.043 | 1.000 |  |
|  | F0Sf-F1Sf==0 | -1.190 | 1.761 | -0.676 | 0.997 |  |
|  | F0Sm-F1Sm==0 | 1.654 | 1.701 | 0.972 | 0.968 |  |
|  | F1Ef-F1Em==0 | -2.154 | 0.585 | -3.682 | 0.018 | ***** |
|  | F1Ef-F1Sf==0 | 1.479 | 1.488 | 0.994 | 0.964 |  |
|  | F1Em-F1Sm==0 | 6.410 | 1.309 | 4.898 | 0.001 | ****** |
|  | F1Sf-F1Sm==0 | 2.777 | 1.893 | 1.467 | 0.775 |  |
|  | **Linear hypotheses** | **Estimate** | **Standard error** | **F value** | **P value** | **Significance level** |
| **Lysozyme1** | Cf-F0Ef==0 | 0.597 | 0.342 | 1.746 | 0.606 |  |
|  | Cf-F0Sf==0 | 2.077 | 0.885 | 2.348 | 0.263 |  |
|  | Cf-F1Ef==0 | -0.994 | 0.274 | -3.630 | 0.021 | ***** |
|  | Cf-F1Sf==0 | -0.284 | 0.612 | -0.464 | 1.000 |  |
|  | Cm-F0Em==0 | 0.552 | 0.308 | 1.791 | 0.577 |  |
|  | Cm-F0Sm==0 | 0.102 | 1.181 | 0.087 | 1.000 |  |
|  | Cm-F1Em==0 | -1.972 | 0.579 | -3.405 | 0.034 | ***** |
|  | Cm-F1Sm==0 | 1.227 | 0.359 | 3.415 | 0.033 | ***** |
|  | F0Ef-F0Em==0 | -0.045 | 0.461 | -0.098 | 1.000 |  |
|  | F0Ef-F0Sf==0 | 1.480 | 0.949 | 1.560 | 0.726 |  |
|  | F0Ef-F1Ef==0 | -1.592 | 0.438 | -3.632 | 0.020 | ***** |
|  | F0Em-F0Sm==0 | -0.450 | 1.221 | -0.368 | 1.000 |  |
|  | F0Em-F1Em==0 | -2.524 | 0.656 | -3.847 | 0.013 | ***** |
|  | F0Sf-F0Sm==0 | -1.975 | 1.476 | -1.338 | 0.849 |  |
|  | F0Sf-F1Sf==0 | -2.361 | 1.076 | -2.195 | 0.336 |  |
|  | F0Sm-F1Sm==0 | 1.124 | 1.235 | 0.911 | 0.979 |  |
|  | F1Ef-F1Em==0 | -0.978 | 0.641 | -1.526 | 0.746 |  |
|  | F1Ef-F1Sf==0 | 0.711 | 0.670 | 1.060 | 0.951 |  |
|  | F0Ef-F1Ef==0 | -1.592 | 0.438 | -3.632 | 0.020 | ***** |
|  | F1Em-F1Sm==0 | 3.199 | 0.681 | 4.694 | 0.002 | ****** |
|  | F1Sf-F1Sm==0 | 1.510 | 0.709 | 2.129 | 0.370 |  |
| **Lysozyme3** | Cf-F0Ef==0 | 1.052 | 0.263 | 3.998 | 0.009 | ****** |
|  | Cf-F0Sf==0 | -1.438 | 0.390 | -3.688 | 0.017 | ***** |
|  | Cf-F1Ef==0 | -0.758 | 0.524 | -1.447 | 0.785 |  |
|  | Cf-F1Sf==0 | 0.136 | 0.650 | 0.210 | 1.000 |  |
|  | Cm-F0Em==0 | 0.279 | 0.622 | 0.449 | 1.000 |  |
|  | Cm-F0Sm==0 | -1.227 | 0.086 | -14.291 | <0.001 | ******* |
|  | Cm-F1Em==0 | -0.947 | 0.177 | -5.365 | <0.001 | ******* |
|  | Cm-F1Sm==0 | 0.451 | 0.107 | 4.212 | 0.006 | ****** |
|  | F0Ef-F0Em==0 | -0.773 | 0.675 | -1.145 | 0.924 |  |
|  | F0Ef-F0Sf==0 | -2.490 | 0.470 | -5.294 | <0.001 | ******* |
|  | F0Ef-F1Ef==0 | -1.810 | 0.586 | -3.088 | 0.062 |  |
|  | F0Em-F0Sm==0 | -1.506 | 0.628 | -2.399 | 0.234 |  |
|  | F0Em-F1Em==0 | -1.227 | 0.646 | -1.897 | 0.498 |  |
|  | F0Sf-F0Sm==0 | 0.211 | 0.399 | 0.529 | 0.999 |  |
|  | F0Sf-F1Sf==0 | 1.574 | 0.758 | 2.077 | 0.390 |  |
|  | F0Sm-F1Sm==0 | 1.678 | 0.137 | 12.226 | <0.001 | ******* |
|  | F1Ef-F1Em==0 | -0.190 | 0.553 | -0.344 | 1.000 |  |
|  | F1Ef-F1Sf==0 | 0.894 | 0.835 | 1.071 | 0.946 |  |
|  | F1Em-F1Sm==0 | 1.398 | 0.207 | 6.772 | <0.001 | ******* |
|  | F1Sf-F1Sm==0 | 0.315 | 0.659 | 0.477 | 1.000 |  |
| **PPO2** | Cf-F0Ef==0 | -2.153 | 0.253 | -8.521 | <0.001 | ******* |
|  | **Linear hypotheses** | **Estimate** | **Standard error** | **F value** | **P value** | **Significance level** |
| **PPO2** | Cf-F0Sf==0 | -0.055 | 1.599 | -0.034 | 1.000 |  |
|  | Cf-F1Ef==0 | -1.753 | 0.673 | -2.604 | 0.166 |  |
|  | Cf-F1Sf==0 | 0.019 | 0.326 | 0.060 | 1.000 |  |
|  | Cm-F0Em==0 | 0.148 | 0.824 | 0.179 | 1.000 |  |
|  | Cm-F0Sm==0 | 0.675 | 1.261 | 0.535 | 0.999 |  |
|  | Cm-F1Em==0 | -2.090 | 0.607 | -3.444 | 0.030 | ***** |
|  | Cm-F1Sm==0 | 0.761 | 0.612 | 1.243 | 0.890 |  |
|  | F0Ef-F0Em==0 | 2.301 | 0.861 | 2.671 | 0.147 |  |
|  | F0Ef-F0Sf==0 | 2.098 | 1.619 | 1.296 | 0.867 |  |
|  | F0Ef-F1Ef==0 | 0.400 | 0.719 | 0.556 | 0.999 |  |
|  | F0Em-F0Sm==0 | 0.527 | 1.506 | 0.350 | 1.000 |  |
|  | F0Em-F1Em==0 | -2.238 | 1.023 | -2.188 | 0.335 |  |
|  | F0Sf-F0Sm==0 | 0.729 | 2.037 | 0.358 | 1.000 |  |
|  | F0Sf-F1Sf==0 | 0.074 | 1.632 | 0.045 | 1.000 |  |
|  | F0Sm-F1Sm==0 | 0.086 | 1.402 | 0.061 | 1.000 |  |
|  | F1Ef-F1Em==0 | -0.337 | 0.906 | -0.372 | 1.000 |  |
|  | F1Ef-F1Sf==0 | 1.773 | 0.748 | 2.370 | 0.250 |  |
|  | F1Em-F1Sm==0 | 2.851 | 0.862 | 3.308 | 0.041 | ***** |
|  | F1Sf-F1Sm==0 | 0.741 | 0.693 | 1.069 | 0.948 |  |

**Supplementary Table 5.** Gene expression analysis of immune genes for *E*. *coli*- (E) and *S*. *entomophila*- (S) infected females (f) and males (m) of the parental (F0) and filial (F1) generation compared to control (C) females and males. Results of **(A)** C_T_s, **(B)** means C_T_ of control females and males, **(C)** ΔΔC_T_ and 2^ΔΔC^_T_**,** and **(D)** their corresponding means, medians, standard deviations (Sd), and standard errors of the mean (Se).

see separate Excel file ‘Supplementary Table5’

**Supplementary Table 6.** Statistical analysis for ΔΔC_T_ data of genes related to histone (de)acetylating and for relative histone acetylation. A parametric multiple comparisons analysis utilizing simultaneous tests for general linear hypotheses on a fitted one way ANOVA model was performed and p-values were adjusted using sandwich estimator. Results for *E*. *coli*- (E) and *S*. *entomophila*- (S) infected females (f) and males (m) of the parental (F0) and filial (F1) generation compared to control (C) females and males. Significance levels: p < 0.05 (*), p < 0.01 (**), p < 0.001 (***). The corresponding raw data can be found in Supplementary Tables 7 and 8.

|  | **Linear hypotheses** | **Estimate** | **Standard error** | **F value** | **P value** | **Significance level** |
| --- | --- | --- | --- | --- | --- | --- |
| **HATchameau** | Cf-F0Ef==0 | -0.413 | 0.560 | -0.738 | 0.994 |  |
|  | Cf-F0Sf==0 | 0.175 | 0.196 | 0.890 | 0.982 |  |
|  | Cf-F1Ef==0 | -0.502 | 0.232 | -2.165 | 0.345 |  |
|  | Cf-F1Sf==0 | 0.492 | 0.045 | 10.884 | <0.001 | ******* |
|  | Cm-F0Em==0 | 0.088 | 0.349 | 0.253 | 1.000 |  |
|  | Cm-F0Sm==0 | 0.300 | 0.316 | 0.949 | 0.973 |  |
|  | **Linear hypotheses** | **Estimate** | **Standard error** | **F value** | **P value** | **Significance level** |
| **HATchameau** | Cm-F1Em==0 | -0.318 | 0.147 | -2.170 | 0.343 |  |
|  | Cm-F1Sm==0 | 0.323 | 0.142 | 2.269 | 0.293 |  |
|  | F0Ef-F0Em==0 | 0.502 | 0.660 | 0.760 | 0.993 |  |
|  | F0Ef-F0Sf==0 | 0.588 | 0.594 | 0.990 | 0.966 |  |
|  | F0Ef-F1Ef==0 | -0.088 | 0.606 | -0.146 | 1.000 |  |
|  | F0Em-F0Sm==0 | 0.212 | 0.471 | 0.450 | 1.000 |  |
|  | F0Em-F1Em==0 | -0.406 | 0.378 | -1.074 | 0.946 |  |
|  | F0Sf-F0Sm==0 | 0.126 | 0.372 | 0.338 | 1.000 |  |
|  | F0Sf-F1Sf==0 | 0.318 | 0.201 | 1.577 | 0.710 |  |
|  | F0Sm-F1Sm==0 | 0.023 | 0.347 | 0.065 | 1.000 |  |
|  | F1Ef-F1Em==0 | 0.184 | 0.274 | 0.671 | 0.997 |  |
|  | F1Ef-F1Sf==0 | 0.994 | 0.236 | 4.208 | 0.006 | ****** |
|  | F1Em-F1Sm==0 | 0.641 | 0.204 | 3.138 | 0.058 |  |
|  | F1Sf-F1Sm==0 | -0.169 | 0.149 | -1.133 | 0.929 |  |
| **HATenoki** | Cf-F0Ef==0 | -0.862 | 0.317 | -2.716 | 0.141 |  |
|  | Cf-F0Sf==0 | -0.006 | 0.323 | -0.020 | 1.000 |  |
|  | Cf-F1Ef==0 | -0.298 | 0.367 | -0.813 | 0.990 |  |
|  | Cf-F1Sf==0 | 0.779 | 0.271 | 2.876 | 0.105 |  |
|  | Cm-F0Em==0 | 0.552 | 0.132 | 4.179 | 0.006 | ****** |
|  | Cm-F0Sm==0 | 0.066 | 0.221 | 0.298 | 1.000 |  |
|  | Cm-F1Em==0 | -0.242 | 0.151 | -1.599 | 0.709 |  |
|  | Cm-F1Sm==0 | 0.279 | 0.142 | 1.968 | 0.475 |  |
|  | F0Ef-F0Em==0 | 1.414 | 0.344 | 4.114 | 0.007 | ****** |
|  | F0Ef-F0Sf==0 | 0.855 | 0.453 | 1.889 | 0.523 |  |
|  | F0Ef-F1Ef==0 | 0.564 | 0.485 | 1.162 | 0.925 |  |
|  | F0Em-F0Sm==0 | -0.486 | 0.257 | -1.889 | 0.524 |  |
|  | F0Em-F1Em==0 | -0.794 | 0.201 | -3.953 | 0.011 | ***** |
|  | F0Sf-F0Sm==0 | 0.072 | 0.391 | 0.185 | 1.000 |  |
|  | F0Sf-F1Sf==0 | 0.786 | 0.422 | 1.864 | 0.539 |  |
|  | F0Sm-F1Sm==0 | 0.213 | 0.262 | 0.811 | 0.990 |  |
|  | F1Ef-F1Em==0 | 0.056 | 0.397 | 0.142 | 1.000 |  |
|  | F1Ef-F1Sf==0 | 1.078 | 0.456 | 2.362 | 0.264 |  |
|  | F1Em-F1Sm==0 | 0.521 | 0.207 | 2.512 | 0.205 |  |
|  | F1Sf-F1Sm==0 | -0.500 | 0.306 | -1.637 | 0.686 |  |
| **HDAC4** | Cf-F0Ef==0 | -0.717 | 0.428 | -1.676 | 0.655 |  |
|  | Cf-F0Sf==0 | 0.057 | 0.487 | 0.116 | 1.000 |  |
|  | Cf-F1Ef==0 | 0.128 | 0.169 | 0.759 | 0.994 |  |
|  | Cf-F1Sf==0 | 1.121 | 0.311 | 3.603 | 0.023 | ***** |
|  | Cm-F0Em==0 | 0.169 | 0.187 | 0.900 | 0.981 |  |
|  | Cm-F0Sm==0 | -0.658 | 0.259 | -2.543 | 0.190 |  |
|  | Cm-F1Em==0 | 0.250 | 0.446 | 0.561 | 0.999 |  |
|  | Cm-F1Sm==0 | 0.938 | 0.326 | 2.873 | 0.102 |  |
|  | **Linear hypotheses** | **Estimate** | **Standard error** | **F value** | **P value** | **Significance level** |
| **HDAC4** | F0Ef-F0Em==0 | 0.886 | 0.467 | 1.897 | 0.513 |  |
|  | F0Ef-F0Sf==0 | 0.774 | 0.648 | 1.194 | 0.912 |  |
|  | F0Ef-F1Ef==0 | 0.845 | 0.460 | 1.838 | 0.551 |  |
|  | F0Em-F0Sm==0 | -0.827 | 0.320 | -2.588 | 0.176 |  |
|  | F0Em-F1Em==0 | 0.082 | 0.484 | 0.169 | 1.000 |  |
|  | F0Sf-F0Sm==0 | -0.715 | 0.551 | -1.297 | 0.871 |  |
|  | F0Sf-F1Sf==0 | 1.064 | 0.578 | 1.842 | 0.548 |  |
|  | F0Sm-F1Sm==0 | 1.596 | 0.417 | 3.831 | 0.014 | ***** |
|  | F1Ef-F1Em==0 | 0.122 | 0.477 | 0.257 | 1.000 |  |
|  | F1Ef-F1Sf==0 | 0.993 | 0.354 | 2.806 | 0.117 |  |
|  | F1Em-F1Sm==0 | 0.687 | 0.553 | 1.243 | 0.894 |  |
|  | F1Sf-F1Sm==0 | -0.183 | 0.451 | -0.406 | 1.000 |  |
| **HDAC6** | Cf-F0Ef==0 | -0.233 | 0.434 | -0.536 | 0.999 |  |
|  | Cf-F0Sf==0 | 0.528 | 0.339 | 1.558 | 0.735 |  |
|  | Cf-F1Ef==0 | -0.569 | 0.446 | -1.275 | 0.883 |  |
|  | Cf-F1Sf==0 | 0.395 | 0.349 | 1.132 | 0.935 |  |
|  | Cm-F0Em==0 | 0.933 | 0.244 | 3.826 | 0.014 | ***** |
|  | Cm-F0Sm==0 | 0.321 | 0.514 | 0.625 | 0.998 |  |
|  | Cm-F1Em==0 | -0.625 | 0.260 | -2.403 | 0.247 |  |
|  | Cm-F1Sm==0 | 0.253 | 0.223 | 1.134 | 0.934 |  |
|  | F0Ef-F0Em==0 | 1.166 | 0.498 | 2.341 | 0.274 |  |
|  | F0Ef-F0Sf==0 | 0.761 | 0.551 | 1.381 | 0.834 |  |
|  | F0Ef-F1Ef==0 | -0.336 | 0.622 | -0.540 | 0.999 |  |
|  | F0Em-F0Sm==0 | -0.612 | 0.569 | -1.076 | 0.950 |  |
|  | F0Em-F1Em==0 | -1.558 | 0.357 | -4.370 | 0.004 | ****** |
|  | F0Sf-F0Sm==0 | -0.207 | 0.616 | -0.336 | 1.000 |  |
|  | F0Sf-F1Sf==0 | -0.134 | 0.487 | -0.275 | 1.000 |  |
|  | F0Sm-F1Sm==0 | -0.069 | 0.560 | -0.122 | 1.000 |  |
|  | F1Ef-F1Em==0 | -0.056 | 0.516 | -0.109 | 1.000 |  |
|  | F1Ef-F1Sf==0 | 0.963 | 0.566 | 1.701 | 0.645 |  |
|  | F1Em-F1Sm==0 | 0.878 | 0.343 | 2.563 | 0.187 |  |
|  | F1Sf-F1Sm==0 | -0.142 | 0.414 | -0.343 | 1.000 |  |
| **SAP18** | Cf-F0Ef==0 | -0.869 | 0.307 | -2.834 | 0.109 |  |
|  | Cf-F0Sf==0 | 0.530 | 0.210 | 2.520 | 0.195 |  |
|  | Cf-F1Ef==0 | 0.154 | 0.274 | 0.561 | 0.999 |  |
|  | Cf-F1Sf==0 | 0.648 | 0.327 | 1.983 | 0.455 |  |
|  | Cm-F0Em==0 | -0.230 | 0.098 | -2.337 | 0.267 |  |
|  | Cm-F0Sm==0 | -0.052 | 0.473 | -0.109 | 1.000 |  |
|  | Cm-F1Em==0 | -0.200 | 0.376 | -0.532 | 0.999 |  |
|  | Cm-F1Sm==0 | 0.194 | 0.265 | 0.733 | 0.995 |  |
|  | F0Ef-F0Em==0 | 0.639 | 0.322 | 1.983 | 0.455 |  |
|  | **Linear hypotheses** | **Estimate** | **Standard error** | **F value** | **P value** | **Significance level** |
| **SAP18** | F0Ef-F0Sf==0 | 1.399 | 0.372 | 3.763 | 0.015 | ***** |
|  | F0Ef-F1Ef==0 | 1.022 | 0.411 | 2.487 | 0.207 |  |
|  | F0Em-F0Sm==0 | 0.179 | 0.483 | 0.369 | 1.000 |  |
|  | F0Em-F1Em==0 | 0.030 | 0.389 | 0.077 | 1.000 |  |
|  | F0Sf-F0Sm==0 | -0.582 | 0.518 | -1.124 | 0.934 |  |
|  | F0Sf-F1Sf==0 | 0.118 | 0.389 | 0.303 | 1.000 |  |
|  | F0Sm-F1Sm==0 | 0.246 | 0.542 | 0.453 | 1.000 |  |
|  | F1Ef-F1Em==0 | -0.354 | 0.465 | -0.760 | 0.993 |  |
|  | F1Ef-F1Sf==0 | 0.495 | 0.426 | 1.160 | 0.922 |  |
|  | F1Em-F1Sm==0 | 0.395 | 0.460 | 0.857 | 0.986 |  |
|  | F1Sf-F1Sm==0 | -0.454 | 0.421 | -1.078 | 0.947 |  |
| **SAP130** | Cf-F0Ef==0 | -0.267 | 0.298 | -0.894 | 0.980 |  |
|  | Cf-F0Sf==0 | 0.287 | 0.204 | 1.409 | 0.802 |  |
|  | Cf-F1Ef==0 | 0.250 | 0.039 | 6.469 | <0.001 | ******* |
|  | Cf-F1Sf==0 | 0.723 | 0.074 | 9.823 | <0.001 | ******* |
|  | Cm-F0Em==0 | 0.376 | 0.063 | 5.925 | <0.001 | ******* |
|  | Cm-F0Sm==0 | 0.209 | 0.329 | 0.637 | 0.998 |  |
|  | Cm-F1Em==0 | -0.133 | 0.266 | -0.499 | 1.000 |  |
|  | Cm-F1Sm==0 | 0.370 | 0.224 | 1.647 | 0.655 |  |
|  | F0Ef-F0Em==0 | 0.643 | 0.305 | 2.106 | 0.367 |  |
|  | F0Ef-F0Sf==0 | 0.554 | 0.361 | 1.533 | 0.728 |  |
|  | F0Ef-F1Ef==0 | 0.517 | 0.301 | 1.717 | 0.609 |  |
|  | F0Em-F0Sm==0 | -0.166 | 0.335 | -0.497 | 1.000 |  |
|  | F0Em-F1Em==0 | -0.509 | 0.274 | -1.857 | 0.517 |  |
|  | F0Sf-F0Sm==0 | -0.078 | 0.387 | -0.201 | 1.000 |  |
|  | F0Sf-F1Sf==0 | 0.436 | 0.217 | 2.012 | 0.421 |  |
|  | F0Sm-F1Sm==0 | 0.160 | 0.398 | 0.403 | 1.000 |  |
|  | F1Ef-F1Em==0 | -0.383 | 0.269 | -1.422 | 0.794 |  |
|  | F1Ef-F1Sf==0 | 0.473 | 0.083 | 5.690 | <0.001 | ******* |
|  | F1Em-F1Sm==0 | 0.502 | 0.348 | 1.443 | 0.783 |  |
|  | F1Sf-F1Sm==0 | -0.353 | 0.236 | -1.496 | 0.751 |  |
| **Relative histone acetylation** | Cf-F0Ef==0 | -49.824 | 29.961 | -1.663 | 0.652 |  |
|  | Cf-F0Sf==0 | 26.351 | 7.324 | 3.598 | 0.014 | ***** |
|  | Cf-F1Ef==0 | -40.322 | 17.874 | -2.256 | 0.282 |  |
|  | Cf-F1Sf==0 | 41.884 | 26.967 | 1.553 | 0.724 |  |
|  | Cm-F0Em==0 | -27.977 | 21.392 | -1.308 | 0.863 |  |
|  | Cm-F0Sm==0 | -56.681 | 38.206 | -1.484 | 0.767 |  |
|  | Cm-F1Em==0 | -87.115 | 35.345 | -2.465 | 0.192 |  |
|  | Cm-F1Sm==0 | -100.822 | 14.703 | -6.857 | <0.001 | ******* |
|  | F0Ef-F0Em==0 | 21.847 | 36.815 | 0.593 | 0.999 |  |
|  | F0Ef-F0Sf==0 | 76.174 | 30.844 | 2.47 | 0.190 |  |
|  | F0Ef-F1Ef==0 | 9.501 | 34.888 | 0.272 | 1.000 |  |
|  | **Linear hypotheses** | **Estimate** | **Standard error** | **F value** | **P value** | **Significance level** |
| **Relative histone acetylation** | F0Em-F0Sm==0 | -28.704 | 43.787 | -0.656 | 0.998 |  |
|  | F0Em-F1Em==0 | -59.138 | 41.315 | -1.431 | 0.798 |  |
|  | F0Sf-F0Sm==0 | -83.031 | 38.902 | -2.134 | 0.347 |  |
|  | F0Sf-F1Sf==0 | 15.533 | 27.944 | 0.556 | 0.999 |  |
|  | F0Sm-F1Sm==0 | -44.141 | 40.938 | -1.078 | 0.948 |  |
|  | F1Ef-F1Em==0 | -46.793 | 39.608 | -1.181 | 0.916 |  |
|  | F1Ef-F1Sf==0 | 82.206 | 32.353 | 2.541 | 0.166 |  |
|  | F1Em-F1Sm==0 | -13.707 | 38.281 | -0.358 | 1.000 |  |
|  | F1Sf-F1Sm==0 | -142.705 | 30.715 | -4.646 | <0.001 | ******* |

**Supplementary Table 7.** Gene expression analysis of genes related to histone (de)acetylating for *E*. *coli*- (E) and *S*. *entomophila*- (S) infected females (f) and males (m) of the parental (F0) and filial (F1) generation compared to control (C) females and males. Results of **(A)** C_T_s, **(B)** means C_T_ of control females and males, **(C)** ΔΔC_T_ and 2^ΔΔC^_T_**,** and **(D)** their corresponding means, medians, standard deviations (Sd), and standard errors of the mean (Se)

see separate Excel file ‘Supplementary Table7’

**Supplementary Table 8.** Relative histone acetylation of *E*. *coli*- (E) and *S*. *entomophila*- (S) infected females (f) and males (m) of the parental (F0) and filial (F1) generation compared to control (C) females and males. Results of **(A)** optical density (OD), **(B)** means OD of control females and males, **(C)** percentage of histone acetlyation and fold histone acetlyation relative to control**,** and **(D)** their corresponding means, medians, standard deviations (Sd), and standard errors of the mean (Se)

see separate Excel file ‘Supplementary Table8’

**Supplementary Table 9.** Statistical analysis for ΔΔC_T_ data of gene related to DNA/RNA methylation and for relative DNA methylation. A parametric multiple comparisons analysis utilizing simultaneous tests for general linear hypotheses on a fitted one way ANOVA model was performed and p-values were adjusted using sandwich estimator. Results for *E*. *coli*- (E) and *S*. *entomophila*- (S) infected females (f) and males (m) of the parental (F0) and filial (F1) generation compared to control (C) females and males. Significance levels: p < 0.05 (*), p < 0.01 (**), p < 0.001 (***).The corresponding raw data can be found in Supplementary Tables 10 and 11.

|  | **Linear hypotheses** | **Estimate** | **Standard error** | **F value** | **P value** | **Significance level** |
| --- | --- | --- | --- | --- | --- | --- |
| **DNMT1** | Cf-F0Ef==0 | -1.241 | 0.309 | -4.014 | 0.009 | ****** |
|  | Cf-F0Sf==0 | 0.070 | 0.232 | 0.303 | 1.000 |  |
|  | Cf-F1Ef==0 | -0.246 | 0.049 | -5.065 | <0.001 | ******* |
|  | Cf-F1Sf==0 | 0.474 | 0.192 | 2.466 | 0.213 |  |
|  | Cm-F0Em==0 | 1.057 | 0.392 | 2.694 | 0.141 |  |
|  | Cm-F0Sm==0 | 0.141 | 0.401 | 0.352 | 1.000 |  |
|  | Cm-F1Em==0 | -0.789 | 0.097 | -8.140 | <0.001 | ******* |
|  | Cm-F1Sm==0 | 0.324 | 0.119 | 2.724 | 0.133 |  |
|  | F0Ef-F0Em==0 | 2.298 | 0.500 | 4.601 | 0.002 | ****** |
|  | **Linear hypotheses** | **Estimate** | **Standard error** | **F value** | **P value** | **Significance level** |
| **DNMT1** | F0Ef-F0Sf==0 | 1.311 | 0.387 | 3.392 | 0.034 | ***** |
|  | F0Ef-F1Ef==0 | 0.995 | 0.313 | 3.181 | 0.053 |  |
|  | F0Em-F0Sm==0 | -0.916 | 0.561 | -1.632 | 0.678 |  |
|  | F0Em-F1Em==0 | -1.846 | 0.404 | -4.568 | 0.003 | ****** |
|  | F0Sf-F0Sm==0 | 0.071 | 0.464 | 0.153 | 1.000 |  |
|  | F0Sm-F1Sm==0 | 0.183 | 0.419 | 0.436 | 1.000 |  |
|  | F1Ef-F1Em==0 | -0.543 | 0.108 | -5.013 | <0.001 | ******* |
|  | F1Ef-F1Sf==0 | 0.719 | 0.198 | 3.631 | 0.020 | ***** |
|  | F1Em-F1Sm==0 | 1.113 | 0.153 | 7.254 | <0.001 | ******* |
|  | F1Sf-F1Sm==0 | -0.150 | 0.226 | -0.663 | 0.997 |  |
| **DNMT2** | Cf-F0Ef==0 | -0.232 | 0.207 | -1.121 | 0.934 |  |
|  | Cf-F0Sf==0 | 0.081 | 0.052 | 1.559 | 0.723 |  |
|  | Cf-F1Ef==0 | 0.050 | 0.068 | 0.741 | 0.994 |  |
|  | Cf-F1Sf==0 | 0.340 | 0.075 | 4.513 | 0.003 | ****** |
|  | Cm-F0Em==0 | -0.256 | 0.420 | -0.608 | 0.998 |  |
|  | Cm-F0Sm==0 | 0.256 | 0.181 | 1.418 | 0.806 |  |
|  | Cm-F1Em==0 | -0.356 | 0.070 | -5.073 | <0.001 | ******* |
|  | Cm-F1Sm==0 | 0.151 | 0.110 | 1.364 | 0.834 |  |
|  | F0Ef-F0Em==0 | -0.024 | 0.469 | -0.050 | 1.000 |  |
|  | F0Ef-F0Sf==0 | 0.313 | 0.214 | 1.466 | 0.779 |  |
|  | F0Ef-F1Ef==0 | 0.282 | 0.218 | 1.296 | 0.867 |  |
|  | F0Em-F0Sm==0 | 0.512 | 0.458 | 1.119 | 0.934 |  |
|  | F0Em-F1Em==0 | -0.100 | 0.426 | -0.236 | 1.000 |  |
|  | F0Sf-F0Sm==0 | 0.175 | 0.188 | 0.933 | 0.976 |  |
|  | F0Sf-F1Sf==0 | 0.259 | 0.091 | 2.835 | 0.107 |  |
|  | F0Sm-F1Sm==0 | -0.105 | 0.212 | -0.498 | 1.000 |  |
|  | F1Ef-F1Em==0 | -0.406 | 0.098 | -4.164 | 0.006 | ****** |
|  | F1Ef-F1Sf==0 | 0.290 | 0.101 | 2.859 | 0.102 |  |
|  | F1Em-F1Sm==0 | 0.507 | 0.131 | 3.872 | 0.012 | ***** |
|  | F1Sf-F1Sm==0 | -0.189 | 0.134 | -1.416 | 0.807 |  |
| **MBD** | Cf-F0Ef==0 | -0.699 | 0.291 | -2.400 | 0.229 |  |
|  | Cf-F0Sf==0 | 0.181 | 0.196 | 0.923 | 0.976 |  |
|  | Cf-F1Ef==0 | -0.292 | 0.211 | -1.383 | 0.816 |  |
|  | Cf-F1Sf==0 | 0.625 | 0.058 | 10.853 | <0.001 | ******* |
|  | Cm-F0Em==0 | 0.367 | 0.015 | 24.410 | <0.001 | ******* |
|  | Cm-F0Sm==0 | -0.001 | 0.307 | -0.002 | 1.000 |  |
|  | Cm-F1Em==0 | -0.698 | 0.208 | -3.359 | 0.034 | ***** |
|  | Cm-F1Sm==0 | 0.436 | 0.149 | 2.935 | 0.083 |  |
|  | F0Ef-F0Em==0 | 1.066 | 0.292 | 3.655 | 0.018 | ***** |
|  | F0Ef-F0Sf==0 | 0.880 | 0.351 | 2.506 | 0.190 |  |
|  | F0Ef-F1Ef==0 | 0.407 | 0.360 | 1.131 | 0.926 |  |
|  | F0Em-F0Sm==0 | -0.368 | 0.307 | -1.196 | 0.904 |  |
|  | **Linear hypotheses** | **Estimate** | **Standard error** | **F value** | **P value** | **Significance level** |
| **MBD** | F0Em-F1Em==0 | -1.065 | 0.208 | -5.111 | <0.001 | ******* |
|  | F0Sf-F0Sm==0 | -0.181 | 0.364 | -0.498 | 1.000 |  |
|  | F0Sf-F1Sf==0 | 0.444 | 0.204 | 2.176 | 0.331 |  |
|  | F0Sm-F1Sm==0 | 0.436 | 0.341 | 1.279 | 0.868 |  |
|  | F1Ef-F1Em==0 | -0.406 | 0.296 | -1.371 | 0.823 |  |
|  | F1Ef-F1Sf==0 | 0.917 | 0.219 | 4.191 | 0.005 | ****** |
|  | F1Em-F1Sm==0 | 1.134 | 0.255 | 4.439 | 0.003 | ****** |
|  | F1Sf-F1Sm==0 | -0.189 | 0.159 | -1.187 | 0.907 |  |
| **Relative DNA methylation** | Cf-F0Ef==0 | 0.500 | 0.101 | 4.945 | 0.006 | ****** |
|  | Cf-F0Sf==0 | 0.068 | 0.330 | 0.206 | 1.000 |  |
|  | Cf-F1Ef==0 | 0.573 | 0.080 | 7.132 | <0.001 | ******* |
|  | Cf-F1Sf==0 | 0.636 | 0.103 | 6.200 | 0.001 | ****** |
|  | Cm-F0Em==0 | 1.147 | 0.093 | 12.276 | <0.001 | ******* |
|  | Cm-F0Sm==0 | 0.058 | 0.101 | 0.573 | 0.998 |  |
|  | Cm-F1Em==0 | 1.145 | 0.096 | 11.891 | <0.001 | ******* |
|  | Cm-F1Sm==0 | 0.973 | 0.093 | 10.409 | <0.001 | ******* |
|  | F0Ef-F0Em==0 | 0.055 | 0.064 | 0.869 | 0.977 |  |
|  | F0Ef-F0Sf==0 | -0.432 | 0.326 | -1.326 | 0.829 |  |
|  | F0Ef-F1Ef==0 | 0.073 | 0.065 | 1.133 | 0.911 |  |
|  | F0Em-F0Sm==0 | -1.089 | 0.040 | -27.442 | <0.001 | ******* |
|  | F0Em-F1Em==0 | -0.002 | 0.026 | -0.086 | 1.000 |  |
|  | F0Sf-F0Sm==0 | -0.602 | 0.322 | -1.867 | 0.519 |  |
|  | F0Sf-F1Sf==0 | 0.568 | 0.327 | 1.740 | 0.593 |  |
|  | F0Sm-F1Sm==0 | 0.915 | 0.040 | 23.069 | <0.001 | ******* |
|  | F1Ef-F1Em==0 | -0.020 | 0.028 | -0.711 | 0.993 |  |
|  | F1Ef-F1Sf==0 | 0.063 | 0.067 | 0.937 | 0.965 |  |
|  | F1Em-F1Sm==0 | -0.173 | 0.026 | -6.706 | <0.001 | ******* |
|  | F1Sf-F1Sm==0 | -0.255 | 0.066 | -3.883 | 0.028 | ***** |

**Supplementary Table 10.** Gene expression analysis of genes related to DNA/RNA methylation for *E*. *coli*- (E) and *S*. *entomophila*- (S) infected females (f) and males (m) of the parental (F0) and filial (F1) generation compared to control (C) females and males. Results of **(A)** C_T_s, **(B)** means C_T_ of control females and males, **(C)** ΔΔC_T_ and 2^ΔΔC^_T_**,** and **(D)** their corresponding means, medians, standard deviations (Sd), and standard errors of the mean (Se)

see separate Excel file ‘Supplementary Table10’

**Supplementary Table 11.** Relative DNA methylation of *E*. *coli*- (E) and *S*. *entomophila*- (S) infected females (f) and males (m) of the parental (F0) and filial (F1) generation compared to control (C) females and males. Results of **(A)** percentage of DNA methylation, **(B)** means of relative DNA methylation of control females and males, **(C) f**old DNA methylation relative to control**,** and **(D)** their corresponding means, medians, standard deviations (Sd), and standard errors of the mean (Se)

see separate Excel file ‘Supplementary Table11’
